# Dermatitis associated with exposure to a marine cyanobacterium during recreational water exposure

**DOI:** 10.1186/1471-5945-8-5

**Published:** 2008-12-30

**Authors:** Nicholas J Osborne, Glen R Shaw

**Affiliations:** 1 Gut and Liver, Murdoch Childrens Research Institute, Flemington Road, Melbourne, Australia; 2 National Research Centre for Environmental Toxicology, The University of Queensland, Brisbane, Australia; 3 Centre for Molecular, Environmental, Genetic and Analytic Epidemiology, University of Melbourne, Australia; 4 School of Public Health, Griffith University, Gold Coast, Australia; 5 Australian Rivers Institute, Griffith University, Brisbane, Australia

## Abstract

**Background:**

Anecdotal evidence reported an outbreak of symptoms on Fraser Island during the late 1990s similar to those expected from exposure to dermotoxins found in the cyanobacterium *L. majuscula*. This coincided with the presence of a bloom of *L. majuscula*.

**Methods:**

Records from the Fraser Island National Parks First aid station were examined. Information on cyanobacterial blooms at Fraser Island were obtained from Queensland National Parks rangers.

**Results:**

Examination of first aid records from Fraser Island revealed an outbreak of symptoms predominantly in January and February 1998.

**Conclusion:**

During a bloom of *L. majuscula *there were numerous reports of symptoms that could be attributed to dermotoxins found in *L. majuscula*. The other four years examined had no *L. majuscula *blooms and the number of *L. majuscula *symptoms was much reduced. These cases comprised a high percentage of the cases treated at the first aid station.

## Background

*Lyngbya majuscula *is a cyanobacterium that has been found to contain highly toxic chemicals including lyngbyatoxin A and debromoaplysiatoxin that cause irritant contact dermatitis [1]. It is common across the globe and large blooms have been reported both in the tropics and subtropics [2]. These may be increasing in frequency as well as an increasing risk of exposure due to increases in the numbers of holiday makers and recreational water users in areas known to have blooms. These have occurred in warmer climates in locations as disparate as Queensland, Australia, Florida, USA and Hawaii, USA. Exposure in humans has been most commonly reported via the dermal route. More rarely exposure via the gastrointestinal tract has been reported [3], including an associated fatality [4]. High rates of severe dermatitis have not been found amongst recreational water users bathing in areas known to be experiencing cyanobacterial blooms [5]. However, periodic outbreaks of dermatitis have been reported in the literature every 10 years or so since the early 1950s [6-10].

Dermatitis caused by *L. majuscula *was recorded in swimmers with symptoms similar to a burn, usually appearing in the genital, perineum and perianal areas. Initial symptoms of erythema and burning sensations, appearing a few hours after exposure gave way to blister formation and deep desquamation, lasting up to several days [6]. Histopathologic examination of human skin exposed to *L. majuscula *found acute, vesicular dermatitis consistent with contact dermatitis on topical application. Microscopic examination revealed superficial desquamation, oedema of the epidermis with vesicles of various sizes within the epidermis (stratum malpighii). Some vesicles contained polymorphonuclear leukocytes and red blood cells and the deepest portion of the epidermis was infiltrated by polymorphonuclear leukocytes. The superficial dermis showed infiltration of both chronic and acute inflammatory cells including mononuclear cells, eosinophils and neutrophils [6] In an animal model a similar set of symptoms and timelines have been observed with the application of cyanobacterial metabolites lyngbyatoxin A and debromoaplysiatoxin [1].

**Figure 1:**
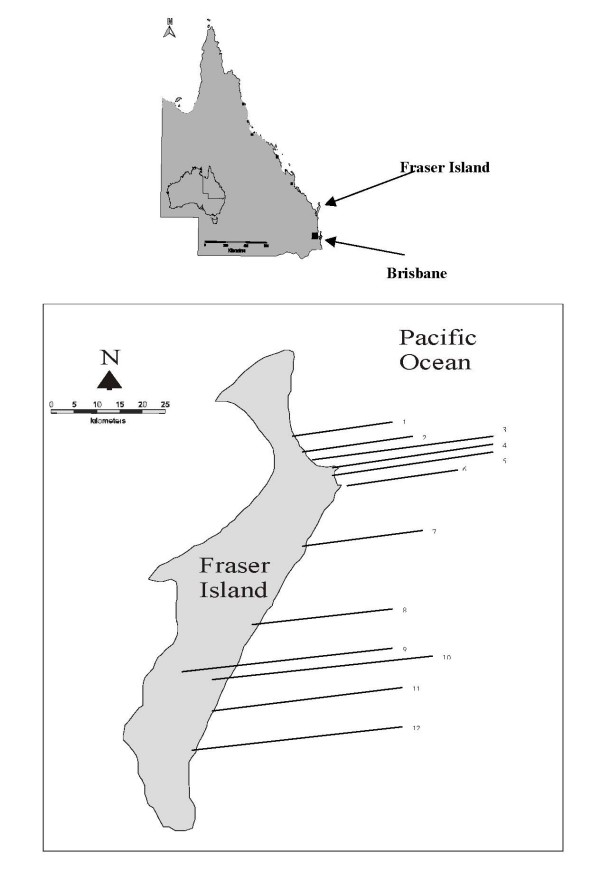
**Location of Fraser Island on the Queensland Coast**. Fraser Island: 1. Ngkala Rocks; 2. Ocean Lake; 3. Orchid Beach; 4. Waddy Point; 5. Champagne Pools; 6. Indian Head; 7. Dundubura; 8. Happy Valley; 9. Lake McKenzie; 10. Lake Wabby; 11. Eurong; 12. Dilli Village.

Fraser Island is the world's largest sand island located 300 km north of Brisbane in South East Queensland, Australia (Figure 1). It is 120 km long and the majority of beaches used for recreation are on the eastern (ocean) side. In recent years there has been anecdotal evidence of blooms of *L. majuscula *and subsequent exposure of marine recreational water users [11]. The aim of this study was to examine first aid records from Fraser Island to ascertain if any deleterious health consequences had a similar timing to outbreaks of *L. majuscula *growth. Furthermore the prevalence of cases of dermatitis amongst clients to the first aid station would be assessed, as would any other factors in order to illuminate whether some subgroups were at greater risk of morbidity.

## Methods

Ethical approval for this study was sought and gained from the Behavioural and Social Sciences Ethical Review Committee, University of Queensland (number B/169/NRCET/SOCPREVMED/99/PHD). Access to first aid records from Fraser Island was obtained by the Freedom of Information Act (Government of Queensland, 1991). Information recorded by the Queensland National Parks Service rangers included date of birth, date of incident, postcode or country of origin of patient, location where treated, sex, occupation, notes on type or symptoms of injury, and medication or action that was taken for the summers of the years 1998–2001. These years were selected as they were around the summer of 1998 when anecdotal cases were reported (Dennison et al., 1999), and as *Lyngbya *was assumed at this stage to be a phenomenon of warmer weather near the summer solstice. Data were collected using Microsoft Excel and analysed with SPSS [12].

Cases of *Lyngbya*-like symptoms were identified subjectively based on the reporting of symptoms in the first aid report. Criteria used to classify symptoms as potentially due to *L. majuscula *in first aid reports of included pain, rash, redness, itching, burning, sore, running nose, sneezing, discharge, swelling, tenderness, slight dry cough, puffiness, inflammation, blister and irritation. These cases were included if they also affected specific tissue or organ of the body including groin, skin, upper respiratory tract and eyes. Some notes also mentioned commencement of symptoms beginning after swimming in sea or exposure to onshore winds. Some cases actually mentioned symptoms were thought to be due to "fireweed" a colloquial term for *L. majuscula *(other colloquialisms also employed are mermaid's hair, dick weed and blanket weed).

## Results

**Figure 2:**
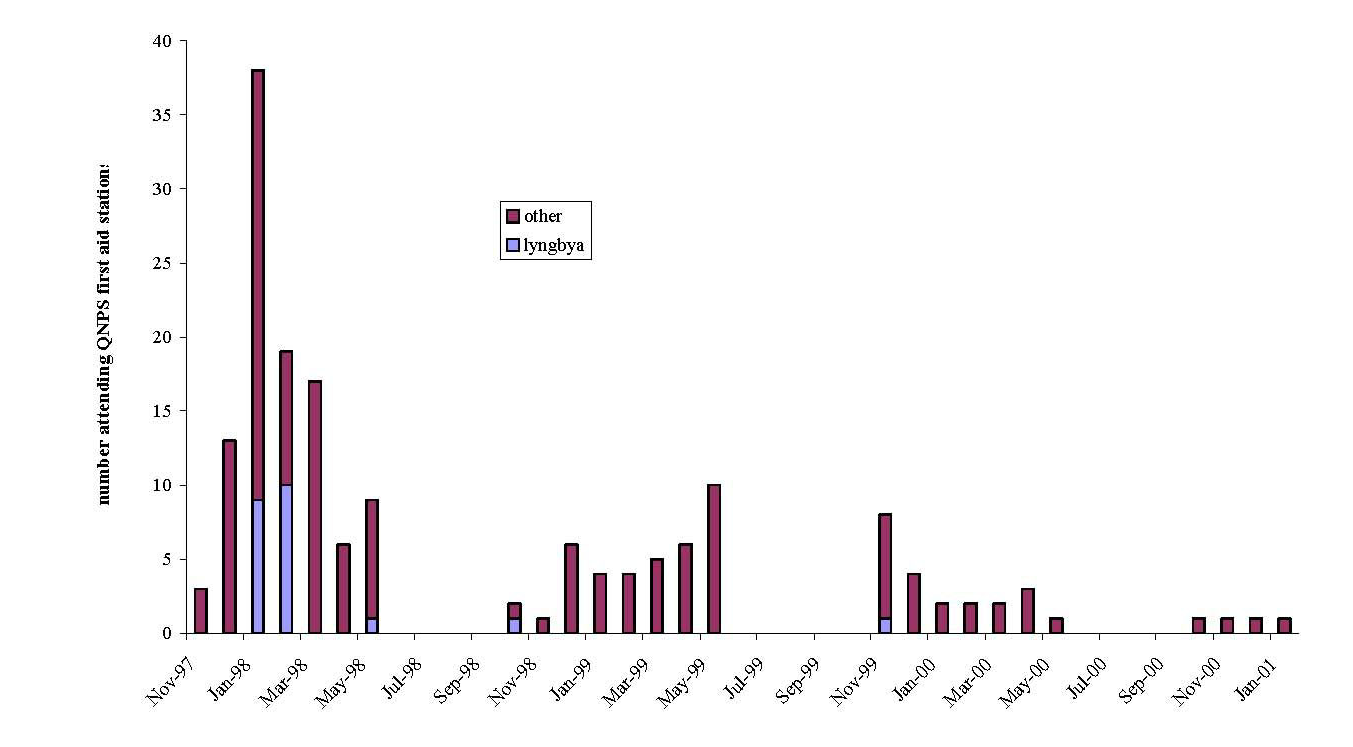
**Reports of *Lyngbya*-like symptoms during the study period**.

There were 176 recorded presentations to first aid stations for the dates shown in Figure 2. The majority (81.0%) of *Lyngbya*-like symptoms occurred in a seven-week period in January and February 1998. Examination of symptoms recorded by Queensland National Parks Service (QNPS) staff of attendees to first aid stations on Fraser Island over the four-year period examined found the highest number were physical injuries. Sixty (44.3%) cases were injuries of a physical nature including reports of injuries sustained in vehicular accidents, broken limbs or sprains. The second highest report was the 21 persons reporting *Lyngbya*-like symptoms (11.9%). Other high numbers of people reported dingo (indigenous Australian wild dog), insect, scorpion and spider bites and burns. Fireweed was mentioned as a potential cause in five of these reports. Other reports excluded as not potentially due to *L. majuscula *exposure included bites, burns (from hot objects) or gastrointestinal problems.

Records of the sex of persons reporting symptoms was predominately not undertaken with only 103 of 176 recording sex. Of the 21 recording *Lyngbya*-like symptoms 3 were recorded as male and 4 female. Due to the low amounts of data on the gender of individuals, no conclusion could be drawn.

Ages of those seeking first-aid treatments ranged from 1 to 71 years old (average 28.4 years). Of these 21 contained symptoms that could be related to exposure to *Lyngbya *with ages ranging from 20 to 48 (average 28.9 years). Seven (33.3%) were treated with ice packs. Other treatments included Loratidine (10 mg/day), 1% Hydrocortizone cream (topically four times daily). No cases required patients to leave the island for further medical assistance.

Injuries with *Lyngbya*-like symptoms were from the Champagne Pools, Eurong and Dundubara on the ocean side of the island (7 missing location). Notably Waddy Point, despite having high numbers of first aid patients recorded (probably due to its popularity and the vicinity to a first aid station), had no *Lyngbya*-like symptoms recorded. Queensland National Parks Service staff were asked of their knowledge of *L. majuscula *and when they had seen or heard of its presence from 1997. Staff reported that it had been present in early 1998 and not afterwards.

Individuals reporting symptoms that could be related to exposure to toxic *L. majuscula *fell into three distinct groups. These included those that reported injury to the groin, the skin on the torso, and face/eyes/respiratory tract. The former two groups were presumed to have had direct exposure to toxic *L. majuscula *(although only one noted symptoms after swimming). The last group were thought to have been exposed to aerosolised *L. majuscula*, perhaps without direct exposure to water. Two individuals mentioned the occurrence of symptoms after driving up the beach (utilised as the main vehicular road and aeroplane landing strip on the island) in sea spray.

## Discussion

The types of symptoms displayed by individuals included in the *Lyngbya*-like symptoms group were similar to those reported by other authors [5,10,13]. In the first aid record notes nine of the twenty-one cases had included injuries that were in the inguinal region. This has been noted as classic symptoms of *Lyngbya*-related exposure whilst swimming [6,14]. Another eight cases reported symptoms common after exposure to aerosolised *L. majuscula *as reported by other authors [9,14]. A further 4 cases reported symptoms of skin itchiness, rash and irritation, similar to those to be expected after marine recreational water activity (MRWA) in waters containing toxic *L. majuscula *[7], and could not be explained by other exposures (see Table 1).

**Table 1:** Reported events of *L. majuscula *toxicity in humans.

**Date**	**Location**	**no. cases**	**author**	**time period**	**maximum period**	**days from solstice**
1958	Oahu, Hawaii	125	Grauer and Arnold, 1961	July–August		+8–70
1959	Oahu, Hawaii		Grauer and Arnold, 1961	late June–July		+8–39
1960	Oahu, Hawaii		Grauer and Arnold, 1961	late June–July		+8–39
1968	Gushikawa, Okinawa	242	Hashimoto *et al.*, 1976	21 July	late afternoon	+29
1976	Oahu, Hawaii		Mynderse *et al.*, 1977	September		
1980	Oahu, Hawaii	86	Serdula *et al.*, 1982	13–26 August	18 August	+57
1983	Maui, Hawaii	31	Anderson *et al.*, 1988	29–31 July	18:00–22:00 hours	+38
1986	Oahu, Hawaii		Izumi and Moore, 1987	summer		

1998	Fraser Island, Australia	21	this study	January-Februrary	February	+47

**Figure 3:**
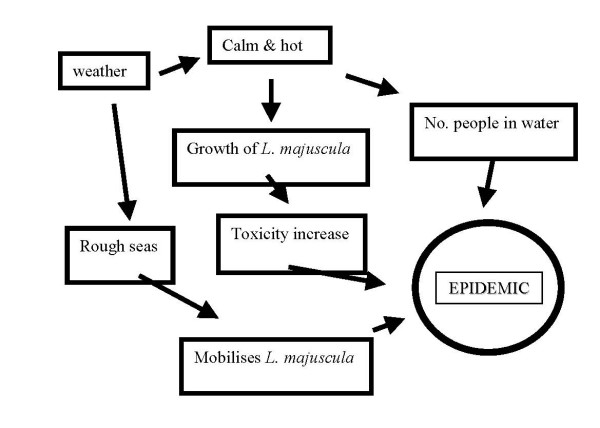
**Conceptual model of exposure to *L. majuscula***.

The vast majority of reports of *L. majuscula*-like symptoms were in a seven-week period of January and February 1998. A third of reported cases occurred on a single day in the middle of this period. This type of short time span of reporting is typical of *L. majuscula *toxic incidents. Of the six outbreaks of dermatitis related to *L. majuscula *reported in the literature, the majority occurred over a small time period during the year. Some reports are limited in information, and only give a range of time periods over a two month period [6], while others are more specific, nominating a single day [14]. Others give a range but nominate a day or two where the majority of cases occur. Reasons for this are two fold, with majority of dermatitic events occurred in a one to two month period after the summer solstice (northern or southern hemisphere, respectively). At this time the seawater has received maximal length of sunlight and began to warm. The *L. majuscula *has received higher amounts of light to grow exponentially. This growth may, in turn, trigger toxin production [15]. Concurrent with the increasing air and water temperatures, increased numbers of individuals are undertaking MRWA. The summer period in subtropical/tropical regions is storm season where may lead to strong onshore winds and water condition where *L. majuscula *is broken up and enters the water column. A combination of these factors leads to an outbreak of dermatitis caused by *L. majuscula*, with the majority of epidemics causing serious injuries effecting larger numbers of people only occurring at a single time at a single location (Figure 3). An alternative hypothesis is that *L. majuscula *growing in Hervey Bay is taken around Sandy Cape by tidal flow and is then picked up by the East Australian current which flows south along the eastern coast of the island. The *L. majuscula *is then subsequently deposited on the ocean beaches of Fraser Island. The rarity of events conspiring towards an outbreak of dermatitis serious enough to warrant visits to health professionals is exemplified by only six events being reported in the literature in 50 years.

Sites where *Lyngbya*-like symptoms were reported were all on the ocean (eastern) side of the island. These included Champagne Pools, Dundubara and Eurong. Several factors would lead to increased incidence of *L. majuscula*-related symptoms on the eastern coast including the presence of *L. majuscula*, presence of individuals undertaking MRWA and meteorological conditions leading to the interaction of these two. The majority of people are on the eastern (ocean) beach on Fraser Island. Much of the western coastline of Fraser Island is not used for recreational purposes. The main avenue of transport from the south to the north of the island is the Seventy-Five Mile Beach (also used as a landing strip for aircraft), on the eastern side of the island. The main settlements on the island are concentrated of this side, including ranger and first aid stations. The coastline on the western side of the island is often swampy and/or populated by mangroves, and has considerable populations of mosquitoes and sand flies. These factors make it unpopular with both the tourist and recreational fishers.

The Champagne Pools, bubbling seawater rock pools, are recommended as a swimming location [16]. It is popular among adults with children for this reason probably due to their calmness, compared to the surf on the ocean beaches, and higher temperature. This is also the location where staff of the Queensland National Parks Service reported *L. majuscula*, and where signs were erected to notify the presence of "harmful algae". Others have reported anecdotal evidence on *L. majuscula*-like symptoms after women and children bathing in 'beach pools', formed by a hump of sand on the beach and pools forming behind them, which were warm during the day [17]. Signs were not erected until April 1998, and in only in one location and hence would not have affected water exposure significantly.

The prevailing winds in the area are south easterly making the eastern side of the island the windward side. Several authors have reported the presence of *L. majuscula *on the windward side of coastlines in diverse geographical locations. Oahu, Hawaii has been noted by several authors in 1958, 1959 and 1960 [6,18] and 1980 [10]. All six beaches where dermatitis has occurred were on the windward side (Laie Bay, Kaawa, Kaneohe Bay, Kailua Bay, Lanikai and Waimanalo Beach) [6]. Toxic incidents purported to have their origin in *L. majuscula *occurred during onshore winds on the island of Maui, Hawaii, soon after tropical storms [9]. Strong winds sweeping over the shore were present during an outbreak of dermatitis linked to *L. majuscula *in Gushikawa Beach, Okinawa in 1968 [7]. A small outbreak of 'seaweed' dermatitis on Oahu in 1986 was on the windward coast [7]. Toxic incidents related to *L. majuscula *occurred on the windward side of Fraser Island (Champagne Pools, Dundubura and Eurong).

'*Lyngbya*' has been reported in Hervey Bay by numerous fishers during the last century [17]. Although the western side of Fraser Island is adjacent to this bay, no reports of *Lyngbya*-like symptoms came from this area by recreational water users. This poses this question that perhaps the increased reporting of *Lyngbya*-like symptoms is due to increased numbers of recreational water users rather than an increase in toxicity, or bloom size and longevity of *L. majuscula*. This increase coincides with increases in Queensland of the population, MRWA, tourism and use of 4-wheel drive vehicles and research into this organism.

## Conclusion

A number of individuals attended Queensland National Parks Service first aid station on Fraser Island with symptoms synonymous with exposure to toxic *L. majuscula*. The majority of these cases occurred in a seven-week period in the summer of 1998. This was also the only period that rangers of the Queensland National Parks Service identified the presence of *L. majuscula *on Fraser Island.

## Competing interests

The authors declare that they have no competing interests.

## Authors' contributions

NO conceived the idea, participated in the design of the study, sourced the data, performed the analysis and drafted the manuscript. GS participated in its design, coordination and writing. All authors read and approved the final manuscript.

## Pre-publication history

The pre-publication history for this paper can be accessed here:


